# Plasma Glial Fibrillary Acidic Protein and N-Terminal Pro B-Type Natriuretic Peptide: Potential Biomarkers to Differentiate Ischemic and Hemorrhagic Stroke

**DOI:** 10.3390/diagnostics13172757

**Published:** 2023-08-25

**Authors:** Eunhee Han, Hyejeong Kim, Bongrae Cho, Jeong-Joong Lee, Soyoung Shin, Eun-Jee Oh, Hyojin Chae

**Affiliations:** 1 Department of Laboratory Medicine, Daejeon St. Mary’s Hospital, College of Medicine, The Catholic University of Korea, Seoul 06591, Republic of Korea; haniyes@catholic.ac.kr (E.H.);; 2 Department of Laboratory Medicine, Seoul St. Mary’s Hospital, College of Medicine, The Catholic University of Korea, Seoul 06591, Republic of Korea

**Keywords:** biomarker, acute stroke, GFAP, NT-proBNP, NF-L, copeptin, ischemic stroke, hemorrhagic stroke

## Abstract

Acute stroke management is critically time-sensitive and challenging. Blood-based biomarkers that can differentiate acute ischemic stroke (IS) from hemorrhagic stroke (HS) can greatly facilitate triage and early management. Admission blood samples obtained within 6 h of stroke symptom onset were analyzed in a derivation/validation design. GFAP, N-FL, NT-proBNP, copeptin, neutrophils (%), NLR, and platelet counts were assessed in the derivation cohort. The informative markers and the derived cutoff values were evaluated in the validation cohort. GFAP > 703 pg/mL showed a PPV of 76.9% and NPV of 95.8% for differentiating HS from IS. Multiple logistic regression analysis showed that GFAP and NT-proBNP were independent variables associated with IS and HS differentiation. Furthermore, applying a combined cutoff (GFAP > 703 pg/mL and NT-proBNP ≤ 125 pg/mL) for HS detection increased the PPV in both the derivation and validation cohorts (93.3% and 100%, respectively). GFAP and NT-proBNP levels were validated as informative blood biomarkers in the differentiation of IS and HS and using a combination of GFAP and NT-proBNP is suggested as a feasible strategy to differentiate stroke subtypes in the hyperacute phase of stroke.

## 1. Introduction

Stoke remains the second leading cause of death worldwide [1], with Asia accounting for approximately two-thirds of the total mortality [2]. In Korea, the age-standardized prevalence, incidence, and mortality rates of stroke are 1.37%, 232 per 100,000 person years, and 29.6 per 100,000 population, respectively, according to a recent publication [3]. Stroke also results in permanent disability for most survivors and is the third leading cause of disability, globally accounting for 5.3% of all disability-adjusted life years (DALYs) lost [4].

Unfortunately, treatment options for acute stoke remains limited, and their efficacy depends on the time between symptom onset and treatment. The two main subtypes of stroke are ischemic stroke (IS) (approximately 85% of all strokes) and hemorrhagic stroke (HS) (remaining 15%) [2], the latter comprising intracerebral hemorrhage (ICH) and subarachnoid hemorrhage. To date, no acute treatment has demonstrated clinical efficacy for ICH; however, early management of patients with acute IS with intravenous thrombolysis or mechanical thrombectomy has proven clinical efficacy. Both therapeutic approaches aim to recanalize occluded vessels and restore blood flow to the ischemic area. Hence, the immediate management of patients with suspected stoke is critical, and the recommended therapeutic windows after symptom onset for intravenous thrombolysis with recombinant tissue plasminogen activator (tPA) and mechanical thrombectomy are <4.5 and <6 h, respectively [5]. Delay in prehospital stroke management is the most important reason for missing recanalization therapy. Recent clinical guidelines support the overarching concept of stroke care involving both prehospital and hospital settings for the effective early management of stroke [5]. The differential diagnosis of diseases presenting with acute stroke-like symptoms relies primarily on clinical assessment and multimodal imaging [6]; however, the limited time frame during which patients are eligible for interventions has spurred interest in blood-based biomarkers and their feasibility in point-of-care testing. If supported by sufficient diagnostic efficacy, blood-based biomarkers of stroke may facilitate prehospital triage before in-hospital multimodal neuroimaging diagnostic techniques can be performed. Moreover, blood-based biomarkers can facilitate the early differentiation of stroke mimics, the frequency of which can be as high as 30% of stroke code activations at the prehospital level [6]. Blood-based biomarkers can provide a reliable, rapid, and cost-effective way to distinguish IS and HS.

The aim of this study was to compare the diagnostic efficacy of selected blood-based biomarkers in differentiating between IS and HS and validate the suggested markers and their cutoff values using a derivation–validation study. To assess the diagnostic efficacy of blood-based biomarkers in the hyperacute phase of stroke, early samples from patients presenting with symptoms suggestive of stroke to a single tertiary hospital within 6 h from symptom onset were enrolled.

## 2. Materials and Methods

### 2.1. Study Population

All stroke patients included in this study were admitted to the emergency department of Seoul St. Mary’s Hospital from October 2021 to February 2023, and all presented within 6 h from the onset of symptoms. Medical records of the patients were retrospectively reviewed, and data regarding patient demographics, final stroke diagnosis, stroke severity, and stroke etiology were retrieved. Stroke diagnosis was determined according to the World Health Organization criteria, based on neurological assessment [7] and neuroimaging results (computed tomography [CT] or magnetic resonance imaging). Stroke severity at admission was assessed using the National Institutes of Health Stroke Scale (NIHSS) score. IS was assessed according to the Trial of Org 10,172 in Acute Stroke Treatment (TOAST) classification when available.

Eligibility was determined based on the following criteria: (1) ≥19 years old; (2) present to the emergency department with suspected stroke; (3) have blood drawn as part of standard care at admission before intravenous thrombolysis; and (4) have sufficient residual samples to facilitate subsequent biomarker measurements. Plasma samples were anticoagulated using ethylenediaminetetraacetic acid and centrifuged at 2000× *g* for 10 min at 20 °C. The residual plasma samples were aliquoted into cryotubes and stored at −80 °C until biomarker measurement.

### 2.2. Study Cohorts

#### 2.2.1. Derivation Cohort

Samples collected between October 2021 and May 2022 were used as a derivation cohort to select the most informative biomarkers and their cut-off values. In the derivation cohort, differences in blood-based biomarkers (neutrophil (%), neutrophil–lymphocyte ratio (NLR) and platelet count, glial fibrillary acidic protein (GFAP), neurofilament light chain (NF-L), N-terminal pro B-type natriuretic peptide (NT-proBNP), and copeptin) between IS and HS were assessed. Additionally, using the area under the receiver operating characteristic curve (AUC) analysis and plotting, ideal cut-off values were derived.

#### 2.2.2. Validation Cohort

Samples collected from June 2022 to February 2023 were used as a validation cohort. Using the validation cohort, the ideal cut-off values of informative markers (that showed statistically significant difference between IS and HS) derived from the derivation cohort were validated.

### 2.3. Measurement of the Biomarkers

GFAP and NF-L levels were measured using single-molecule array (SiMoA) methodology, as previously described [8]. Measurements were performed using the Human Neurology 2-Plex B assay kit on the SIMOA HD-1 analyzer (Quanterix, Billerica, MA, USA), according to the manufacturer’s instructions. Samples were analyzed at a 4-fold dilution, measured in duplicate, and the mean of each test was used in the statistical analysis. The corresponding inter-assay coefficients of variability (CVs) for the GFAP, as determined using internal quality controls for low (116.7 pg/mL) and high (1145 pg/mL) concentrations, were 6.7% and 6.5%, respectively. For NF-L, the inter-assay CVs of internal quality controls for low (7.79 pg/mL) and high (722 pg/mL) concentrations were 3.1% and 6.4%, respectively.

NT-proBNP was measured using an electrochemiluminescence immunoassay using the Elecsys proBNP II reagent (Roche Diagnostic, Mannheim, Germany) on the cobas e 801 immunoassay analyzer (Roche). Copeptin was measured using a colorimetric immunoassay using a human copeptin ELISA kit (Novus Biologicals, UK) and the SpectraMax 190 Absorbance Microplate Reader (Molecular Devices LLC, San Jose, CA, USA), according to the manufacturer’s instructions. All measurements were performed without knowledge of the patients’ clinical diagnoses.

Other blood-based biomarkers (neutrophil (%), neutrophil–lymphocyte ratio (NLR), and platelet count) were retrieved from the medical records. The study protocol was approved by the Institutional Review Board (IRB) and Ethics Committee of Seoul St. Mary’s Hospital.

### 2.4. Statistical Analysis

Categorical variables are expressed as numbers (percentages), and continuous variables are expressed as medians and interquartile ranges (IQRs) for non-normally distributed data. Normality was assessed using the D’Agostino–Pearson normality test, and because most variables did not follow a normal distribution, medians were used for intergroup comparisons between patients with IS and HS.

In the derivation cohort, univariate analyses were performed using the chi-squared test and Mann–Whitney U test for categorical and continuous variables, respectively. A logistic regression model was developed using statistically significant variables on univariate analysis (*p* value < 0.05) from the derivation cohort. AUC analysis was performed for each biomarker and biomarker combination. Suggestions for optimal cutoff values were derived from AUC analysis and plotting. Statistical analyses were performed using MedCalc (version 20.111; MedCalc Software Ltd., Ostend, Belgium) and SAS (version 9.4; SAS Institute, Inc., Cary, NC, USA), and a two-tailed *p* value < 0.05 was considered statistically significant.

## 3. Results

### 3.1. Derivation Cohort

In the derivation cohort, 73 samples from patients with acute stroke-like symptoms were included; the final clinical diagnoses were IS in 51 patients (69.9%) and HS in 22 patients (30.1%). The baseline characteristics of the derivation cohort are presented in Table 1. The patients diagnosed with IS had a higher median age than those diagnosed with HS (73 vs. 55 years, *p* < 0.001). The patients with IS had a higher frequency of atrial fibrillation (AF) and hypertension (*p* = 0.001 and *p* = 0.004, respectively) than those with HS. The median last known well (LKW) to sampling time was 188 (IQR: 85–597) min. There was no significant difference in the median sampling time from the LKW between the IS and HS groups.

The GFAP (IQR) concentration was significantly higher in patients with HS compared with IS [2171.0 (922.8–5883.0) vs. 213.8 (161.8–439.4) pg/mL, *p* < 0.001; Table 2], whereas, the NT-proBNP (IQR) concentration was significantly lower in patients with HS compared with IS [44.2 (14.8–209.0) vs. 312.0 (85.8–1206.0) pg/mL, *p* < 0.001]. No significant intergroup differences were found for NF-L and copeptin concentrations, or other biomarkers including neutrophils (%), neutrophil-to-lymphocyte ratio (NLR), and platelet counts. When statistically significant univariate variables were included in the logistic regression analysis, only GFAP and NT-proBNP remained in the model and were independently associated with IS and HS differentiation (Table 3). The AUC for differentiating patients with IS and HS was 0.886 (95% CI: 0.790–0.948) for GFAP and 0.775 (95% CI: 0.662–0.864) for NT-proBNP (Table 4). Additionally, the cutoff values that offered the best sensitivity and specificity for differentiating HS from IS were identified as GFAP > 703 pg/mL (sensitivity 90.9%, specificity 88.2%), and NT-proBNP ≤ 292 pg/mL (sensitivity 90.9%, specificity 52.9%) from the derivation cohort (Table 4).

**Figure 1:**
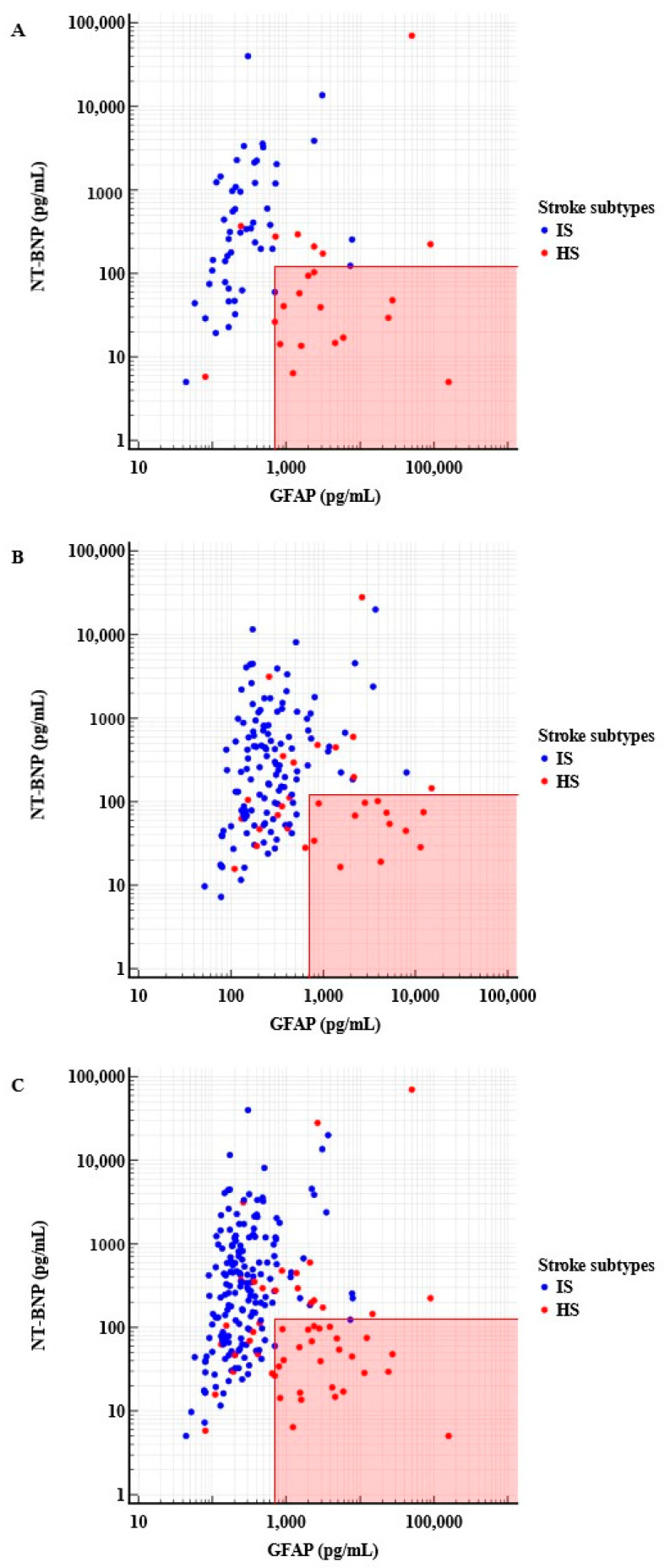
Plots demonstrating the differentiation of IS and HS by utilizing both GFAP (*x* axis, pg/mL) and NT-proBNP concentrations (*y* axis, pg/mL), with positivity values of both GFAP > 703 pg/mL and NT-proBNP ≤ 125 pg/mL in the area highlighted red. (**A**) Patients in the derivation cohort (IS = 51, HS = 22). (**B**) Patients in the validation cohort (IS = 122, HS = 54). (**C**) Patients in total cohorts (IS = 173, HS = 54).

Plotting GFAP and NT-proBNP together revealed that a combined value might detect HS with improved specificity (Figure 1). On visual inspection of the scatter plot, patients with HS generally had GFAP concentrations > 703 pg/mL together with NT-proBNP concentrations ≤ 125 pg/mL. These combined cut-off values enabled HS detection with 63.6% sensitivity and 98% specificity (PPV, 93.3%; NPV: 86.2%) (Table 4 and Table S3).

### 3.2. Validation Cohort

The validation cohort consisted of 154 samples (122 IS and 54 HS). There were no significant differences in baseline characteristics between the derivation and the validation cohort (Table S1; the baseline characteristics of the whole cohort are detailed in Table S2). Of the multiple biomarkers assessed in the derivation cohort, only the two informative markers, GFAP and NT-proBNP, were measured in the validation cohort. As in the derivation cohort, the median GFAP (IQR) concentration was significantly higher in patients with HS compared with IS [1077 (362.5–3360.0) vs. 238 (152.0–383.0) pg/mL, *p* < 0.001], whereas the median NT-proBNP (IQR) concentration was significantly lower in patients with HS compared with IS [75.4 (45.1–184.5) vs. 253 (70.4–795) pg/mL, *p* = 0.002]. In addition, there were no significant differences in the GFAP and NT-proBNP levels for both HS and IS subgroups between the derivation and the validation cohort. Additional logistic regression analysis of the whole cohort repeatedly demonstrated GFAP and NT-proBNP as independent variables of IS and HS differentiation (Table 3). In the validation cohort, the AUCs for differentiating patients with HS and IS were 0.803 (95% CI: 0.732–0.863) for GFAP and 0.679 (95% CI: 0.599–0.752) for NT-proBNP. When the individual cut-off values derived from the derivation cohort were applied to the validation cohort, GFAP > 703 pg/mL and NT-proBNP ≤ 292 pg/mL each showed slight decrease in PPV (61.3% and 27.0%, respectively), but comparable NPV (89.4% and 89.1%, respectively) as in the derivation cohort. Furthermore, the combined cutoff values of GFAP > 703 pg/mL and NT-proBNP ≤ 125 pg/mL enabled relatively specific detection of HS (PPV 100%, NPV 86.5%) in the validation cohort (Table 4).

## 4. Discussion

This study assessed the diagnostic efficacy of selected blood-based biomarkers, including GFAP, NF-L, NT-proBNP, copeptin, neutrophils (%), NLR, and platelet counts, in differentiating IS and HS in the hyperacute phase of stroke. From the derivation cohort, the GFAP and NT-proBNP levels were identified as informative biomarkers in distinguishing IS and HS in patients presenting within 6 h of symptom onset, while NF-L, copeptin, and other markers did not show clinical value.

GFAP is an intermediate filament found exclusively in astrocytes [9]; the differential levels of GFAP between IS and HS in the hyperacute phase of stroke shown in this study validate previous reports [10,11,12]. While the mechanisms of GFAP release at the cellular and tissue levels are still to be investigated, the diagnostic performance of GFAP in distinguishing IS and HS can be attributed to the different kinetics of astrocytic cell death. Necrosis ensues within the first few hours of symptom onset in HS, while astrocytic cell death peaks between 48 and 98 h after onset in IS; this is revealed by the release of GFAP into the peripheral circulation [13].

In contrast, NF-L is a neuronal intermediate filament that is a major component of the axonal cytoskeleton [14]. NF-L is released into the extracellular space, crosses the blood–brain barrier, and can be detected in peripheral blood upon acute and chronic axonal injury depending on the extent of damage [15]. To the best of our knowledge, none of the previous studies have compared NF-L levels between IS and HS subtypes in the hyperacute phase, and although the median NF-L levels were similar to the previously reported NF-L concentrations in either IS (16 pg/mL) or HS (19.8 pg/mL) patients [14,15], our study demonstrates that the NF-L is a nonviable marker for differentiating between IS and HS in the hyperacute phase. The NF-L levels in IS were previously reported to peak between week 3 and 30 days [16,17], although the release dynamics in HS remain to be determined.

Although not a brain-specific marker, NT-proBNP levels have been suggested as an optimal biomarker for cardioembolic stroke diagnosis in the acute phase [18,19,20,21]; our data demonstrate that NT-proBNP is a significant variable in differentiating IS and HS in the hyperacute phase of stroke. From a subgroup analysis of IS patients, NT-proBNP concentrations were significantly higher in the cardioembolism subtype than in any other etiologic subtypes of IS according to the TOAST classification (Figure S1). Moreover, on logistic regression analysis, the NT-proBNP level was independently associated with the differentiation of IS and HS, even after adjustment for age, sex, AF, cardiovascular risk factors, and stroke severity.

In acute stroke management, tPA is the treatment of choice for IS, after excluding ICH, and the benefits of tPA are time dependent, with the early initiation of a tPA resulting in better outcomes [22]. However, to safeguard against the risk of giving a tPA to a patient with ICH, especially if prehospital POC assays become available, a high level of specificity in diagnosing IS is mandatory. Importantly, the biomarkers proposed should at least increase the specificity rates close to 100% to safely mitigate the possibility of administering tPA to patients with ICH [23]. This study has shown that the combined cutoff values of GFAP and NT-proBNP for HS can increase the specificity of either biomarker alone. Although this finding needs to be validated in future studies, applying GFAP and NT-proBNP jointly in this study resulted in an increase in specificity for the diagnosis of HS compared with the specificity achieved with GFAP or NT-proBNP alone.

Nevertheless, the results of this study should be interpreted with caution, considering the following limitations. The number of patients recruited for this study was relatively small. Therefore, further studies are required to validate the gain in specificity by combining GFAP and NT-proBNP levels in larger patient cohorts. Furthermore, we did not perform serial biomarker measurements. Therefore, we were unable to collect data on the dynamic pattern of each biomarker, which might have provided additional information regarding its diagnostic performance. Finally, we are aware that variability among different GFAP assays may affect diagnostic accuracy, and to justify the implementation of GFAP in prehospital POC testing, improvements in the sensitivity of POC assays will be mandatory.

## 5. Conclusions

In conclusion, our study confirms the role of GFAP and NT-proBNP in the differentiation of IS and HS during the hyperacute stage of stroke. Additionally, this study provides compelling evidence of combining GFAP with NT-proBNP to improve diagnostic specificity and exclude ICH. Conversely, NF-L, copeptin, and NLR did not demonstrate clinical utility in differentiation between IS and HS. The measurements of blood GFAP and NT-proBNP assessment represent a relatively simple method and can significantly improve the discriminatory accuracy diagnosis of stroke subtypes in particular, in an emergency environment where imaging equipment is not provided. Future studies involving larger cohorts are required to validate the GFAP and NT-proBNP combined algorithm; however, our study validates the diagnostic utility of blood-based biomarkers in the differentiation of IS and HS in the hyperacute phase of stroke.

## Figures and Tables

**Table 1:** Baseline clinical characteristic of patients in the derivation cohort and comparison between those with ischemic vs. hemorrhagic stroke.

	Derivation Cohort			
	All	IS	HS	*p* Value
Number of patients	73	51	22	
Age (years), median (IQR)	72 (59–79)	73 (68–83)	55 (48–72)	<0.001 †
Female, *n* (%)	39 (53.4)	31 (60.8)	8 (36.4)	0.218 ‡
Medical history, *n* (%)				
Atrial fibrillation, *n* (%)	18 (24.7)	17 (33.3)	1 (4.5)	0.001 ‡
Diabetes, *n* (%)	17 (23.3)	15 (29.4)	2 (9.1)	0.061 ‡
Dyslipidemia, *n* (%)	16 (21.9)	13 (25.5)	3 (13.6)	0.265 ‡
Hypertension, *n* (%)	42 (57.5)	35 (68.6)	7 (31.8)	0.004 ‡
Ischemic heart disease, *N* (%)	5 (6.8)	5 (9.8)	0 (0)	0.130 ‡
IS history, *n* (%)	15 (20.5)	13 (25.5)	2 (0.8)	0.114 ‡
ICH history, *n* (%)	3 (4.1)	2 (3.9)	1 (4.5)	0.907 ‡
LKW to sample time (min), median (IQR)	188 (85–597)	278 (101–633)	131 (60–527)	0.114 †
<1 h, *n* (%)	9 (12.3)	4 (7.8)	5 (22.7)	0.078 ‡
<3 h, *n* (%)	36 (49.3)	23 (45.1)	13 (59.1)	0.276 ‡

† Mann–Whitney U test; ‡ Chi-square tests; Abbreviation: IS, ischemic stroke; HS, hemorrhagic stroke; ICH, intracerebral hemorrhage; LKW, last known well.

**Table 2:** Intergroup differences in GFAP, NT-proBNP, NF-L, copeptin, neutrophil (%), NLR, and platelet count in the derivation cohort.

	Ischemic Stroke			Hemorrhagic Stroke			
Biomarker	Number of Patients	Median	IQR	Number of Patients	Median	IQR	*p* Value †
GFAP (pg/mL)	51	213.8	161.8–439.4	22	2171.0	922.8–5883	**<0.001**
NT-proBNP (pg/mL)	51	312	85.8–1206	22	44.2	14.8–209	**<0.001**
NF-L (pg/mL)	51	26.6	17.0–35.7	22	26.2	12.3–49.8	0.848
Copeptin (pg/mL)	49	41.1	26.4–81.2	21	41.9	22.0–64.7	0.715
Neutrophil %	51	65.6	57.9–76.1	22	60.1	46.4–71.7	0.161
NLR	51	2.7	1.7–4.8	22	2.0	1.1–3.6	0.136
Platelet (10^9^/L)	51	226	185.3–278.3	22	257.0	222.0–283.0	0.102

† Mann–Whitney U test. Abbreviations: IQR, interquartile range; GFAP, glial fibrillary acidic protein, NF-L, neurofilament light chain; LKW, last known well; NT-proBNP, N-terminal pro B-type natriuretic peptide; NLR, neutrophil-to-lymphocyte ratio.

**Table 3:** Logistic regression analysis for comparison between ischemic and hemorrhagic stroke in the derivation and the whole cohort.

	Derivation Cohort		Whole Cohort	
	Coefficient	*p* Value	Coefficient	*p* Value
Age	0.01517	0.359	0.01313	0.490
Atrial fibrillation	−0.5149	0.204	−0.38441	0.375
Hypertension	−0.34524	0.151	−0.14318	0.607
GFAP	−0.00057	**<0.001**	−0.00064	**<0.001**
NT-proBNP	0.00033	**0.030**	0.00038	**0.016**

Abbreviations: 95% CI, 95% confidence interval; GFAP, glial fibrillary acidic protein; NT-proBNP, N-terminal pro B-type natriuretic peptide.

**Table 4:** Performance of GFAP and NT-proBNP for differentiating ischemic vs. hemorrhagic stroke in the derivation and the validation cohort.

Biomarker	Derivation Cohort							Validation Cohort			
	AUC (95% CI)	*p*	Ideal Cut-Off	Sensitivity% (95% CI)	Specificity% (95% CI)	PPV (95% CI)	NPV (95% CI)	Sensitivity% (95% CI)	Specificity% (95% CI)	PPV (95% CI)	NPV (95% CI)
GFAP	0.886 (0.790–0.948)	<0.001	>703	90.9 (70.8–98.9)	88.2 (76.1–95.6)	76.9 (60.9–87.7)	95.8 (85.7–98.8)	59.4 (40.6–76.3)	90.2 (83.4–94.8)	61.3 (46.3–74.4)	89.4 (84.7–92.8)
NT-BNP	0.775 (0.662–0.864)	<0.001	≤292	90.9 (70.8–98.9)	52.9 (38.5–67.1)	45.5 (37.7–53.4)	93.1 (77.8–98.1)	77.4 (58.9–90.4)	46.7 (37.6–56.0)	27.0 (22.3–32.2)	89.1 (80.5–94.1)
GFAP and NT-BNP	0.781 (0.669–0.870)	<0.0001	>703 (GFAP) and ≤125 (NT-BNP)	95.5 (77.2–99.9)	60.8 (46.11–74.16)	93.3 (66.2–99.0)	86.2 (78.2–91.6)	38.7 (21.7–57.8)	100 (97.0–100)	100 (73.5–100)	86.5 (82.9–89.5)

Abbreviations: AUC, area under curve; CI, confidence interval; GFAP, glial fibrillary acidic protein; BNP, B-type natriuretic peptide; LKW, last known well; PPV, positive predictive value; NPV, negative predictive value.

## Data Availability

The data presented in this study are available upon request from the corresponding author.

## Supplementary Materials

The following supporting information can be downloaded at: https://www.mdpi.com/article/10.3390/diagnostics13172757/s1, Figure S1: NT-proBNP levels according to the etiologic subtype classification of ischemic stroke; Table S1: Clinical characteristics of patients in the derivation and validation cohorts; Table S2: Baseline characteristics of the whole cohort and comparison between ischemic and hemorrhagic stroke; Table S3: Performance of GFAP and NT-proBNP for differentiating ischemic vs. hemorrhagic stroke in the derivation and the validation cohort.
