# Supplementary material for: Plasma Glial Fibrillary Acidic Protein and N-Terminal Pro B-Type Natriuretic Peptide: Potential Biomarkers to Differentiate Ischemic and Hemorrhagic Stroke
Source: Diagnostics (Basel). 2023 Aug 25;13(17):2757. doi: 10.3390/diagnostics13172757 (PMC10486392; doi:10.3390/diagnostics13172757)
Supplement: Supplementary file 1 [file diagnostics-13-02757-s001.zip › diagnostics-2493257-supplementary.pdf]

Table S1. Clinical characteristic of patients in the derivation and validation cohorts.

Variable	All	Derivation cohort	Validation cohort	*p* Value
Number of patients	227	73	154	
Age (yr), median (IQR)	72.0 (62.0-80.0)	72.0 (58.8-79.5)	73.0 (62.0-80.0)	0.479†
Female, n (%)	104 (45.8)	39 (53.4)	65 (42.2)	0.114‡
Medical history, n (%)				
Atrial fibrillation	53 (23.3)	18 (24.7)	35 (22.7)	0.749‡
Hypertension	134 (59.0)	42 (57.5)	92 (59.7)	0.753‡
Ischemic heart disease	22 (9.7)	5 (6.8)	17 (11.0)	0.320‡
Diabetes	63 (27.8)	17 (23.3)	46 (29.9)	0.302‡
Dyslipidemia	56 (24.7)	16 (21.9)	40 (26.0)	0.509‡
IS history	41 (18.1)	15 (20.5)	26 (16.9)	0.504‡
ICH history	12 (5.3)	3 (4.1)	9 (5.8)	0.756*
NIHSS at admission[a], median (IQR)	4.0 (1.0-10.5)	5.5 (2.0-12.0)	4.0 (1.0-10.0)	0.069‡
TOAST, n (%)				
Large artery atherosclerosis	65 (28.6)	17 (23.3)	48 (36.2)	
Cardioembolic	50 (22.0)	18 (24.7)	32 (20.8)	
Small vessel occlusion	31 (13.7)	8 (11.0)	23 (14.9)	
Undetermined	21 (9.3)	7 (9.6)	14 (9.1)	
LKW to sample time, minutes, median (IQR)	207.0 (80.5-631.8)	188.0 (84.8-605.3)	215.0 (72.5-672.5)	0.832†
<1 h, n (%)	48 (21.1)	9 (12.3)	39 (25.3)	0.025‡
<3 h, n (%)	114 (50.2)	36 (49.3)	78 (50.6)	0.851‡

IS, ischemic stroke; HS, hemorrhagic stroke; ICH, intracerebral hemorrhage; TOAST, Trial of Org 10172 in Acute Stroke Treatment; LKW, last known well; FAT, first abnormal time.

[a]NIHSS score was only available in patients with ischemic stroke.

† Mann-Whitney U tests; ‡ Chi-square tests; * Fisher's exact test.

Table S2. Baseline characteristics of the whole cohort and comparison between ischemic vs hemorrhagic stroke

	Total	Ischemic stroke	Hemorrhagic stroke	*P* Value
Number of patients	227	173	54	
Age (years), median (IQR)	72 (62-80)	73 (64-81)	67 (51-75)	**0.002†**
Female, N (%)	104 (45.8)	81 (46.8)	23 (42.6)	0.587‡
Medical history, N (%)				
Atrial fibrillation, N (%)	53 (23.4)	51 (29.5)	2 (3.7)	**<0.001‡**
Diabetes, N (%)	63 (27.8)	53 (30.6)	10 (18.5)	0.083‡
Dyslipidemia, N (%)	56 (24.7)	48 (27.7)	8 (14.8)	0.055‡
Hypertension, N (%)	134 (59.0)	105 (60.7)	29 (53.7)	0.363‡
Ischemic heart disease, N (%)	22 (9.7)	20 (11.6)	2 (3.7)	0.089‡
IS history, N (%)	41 (18.1)	37 (21.4)	4 (7.4)	**0.020‡**
ICH history, N (%)	12 (5.3)	5 (3.0)	7 (13.0)	**0.009***
LKW to sample time (min), median (IQR)	188 (85-597)	278.0 (101.3-633.0)	131.0 (60.0-527.0)	0.114†
<1 h, N (%)	48 (21.1)	32 (17.3%)	16 (29.6%)	0.081‡
<3 h, N (%)	114 (50.2)	80 (46.2%)	34 (63.0%)	**0.032‡**

IS, ischemic stroke; HS, hemorrhagic stroke; ICH, intracerebral hemorrhage; LKW, last known well.

† Mann-Whitney U tests; ‡ Chi-square tests; * Fisher's exact test.

Table S3. Performance of GFAP and NT-proBNP for differentiating ischemic vs. hemorrhagic stroke in the derivation and the validation cohort

			Derivation cohort				Validation cohort				Total cohort			
Biomarker	AUC (95% CI)	Ideal cut-off	Sensitivity % (95% CI)	Specificity % (95% CI)	PPV (95% CI)	NPV (95% CI)	Sensitivity % (95% CI)	Specificity % (95% CI)	PPV (95% CI)	NPV (95% CI)	Sensitivity % (95% CI)	Specificity % (95% CI)	PPV (95% CI)	NPV (95% CI)
GFAP	0.886 (0.790 - 0.948)	>703	90.9 (70.8-98.9)	88.2 (76.1-95.6)	76.9 (60.9-87.7)	95.8 (85.7-98.8)	59.4 (46.7-76.3)	90.2 (83.5-94.8)	61.3 (46.3-74.4)	89.4 (84.7-92.8)	72.2 (58.4-83.5)	89.6 (84.1-93.7)	68.4 (57.6-77.6)	91.2 (87.0-94.1)
NT-proBNP	0.775 (0.662 - 0.864)	≤292	90.9 (70.8-98.9)	52.9 (38.5-67.1)	45.5 (37.7-53.4)	93.1 (77.8-98.1)	77.4 (58.9-90.4)	46.7 (37.6-56.0)	27.0 (22.3-32.2)	89.1 (80.5-94.1)	83 (72.0-91.9	48.6 (40.9-56.3)	33.1 (29.0-37.4)	90.3 (83.5-94.5)
GFAP and NT-proBNP	0.808 (0.699 - 0.891)	>703(GFAP), ≤125(NT-BNP)	63.6 (40.7-82.8)	98.0 (89.6-100)	93.3 (66.2-99.0)	86.2 (78.2-91.6)	38.7 (21.9-57.8)	100 (97.0-100)	100 (73.5-100)	86.5 (82.9-89.5)	49.1 (35.1-63.2)	99.4 (96.8-100)	96.3 (78.3-99.5)	86.4 (83.0-89.3)

AUC, area under curve; CI, confidence interval; GFAP, glial fibrillary acidic protein; NT-proBNP, N-terminal pro B-type natriuretic peptide; LKW, last known well; PPV, positive predictive value; NPV, negative predictive value.

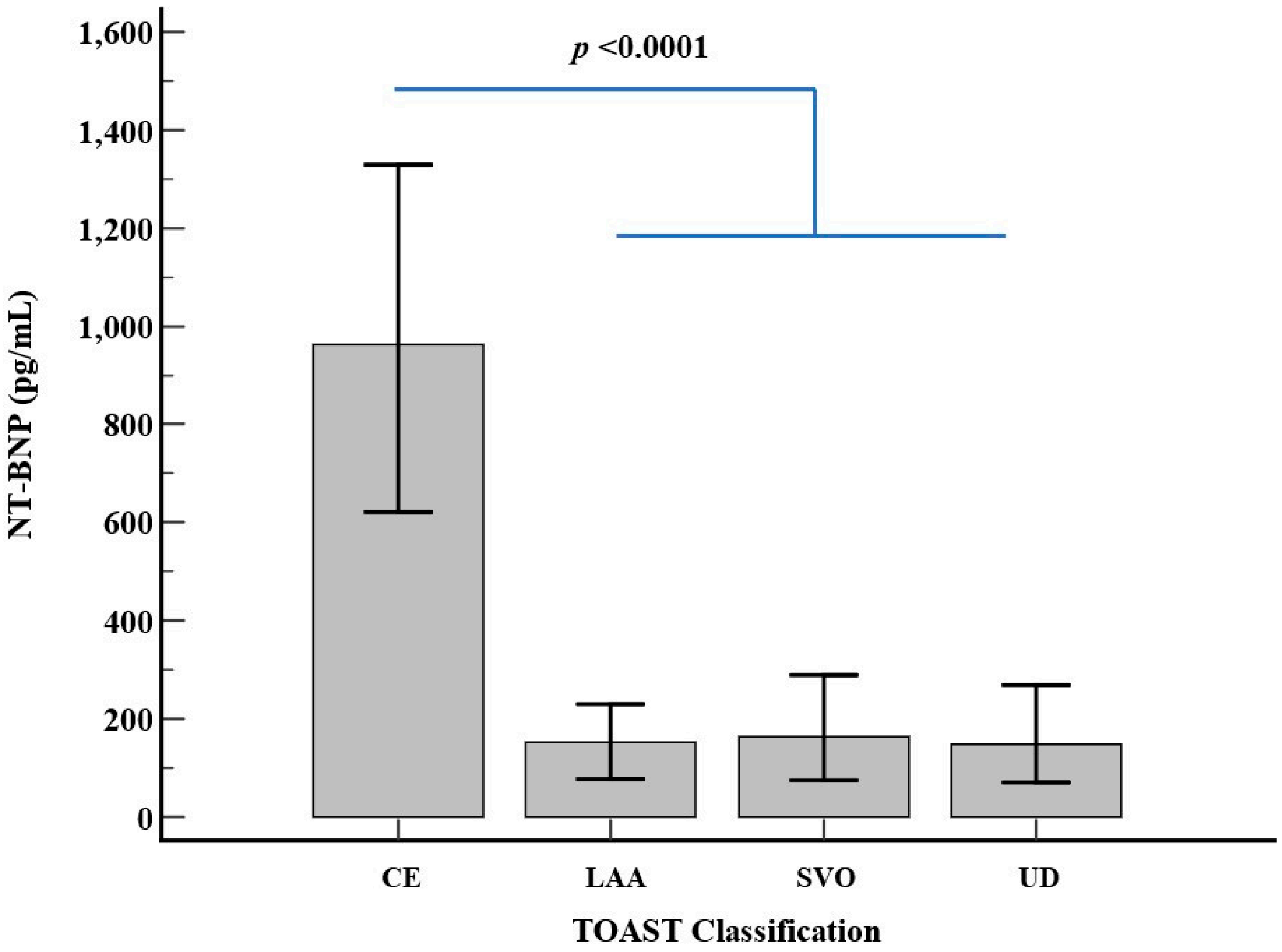

Figure S1. NT-proBNP levels according to the etiologic subtype classification of ischemic stroke (n = 173). Among the etiological subtypes of IS, NT-proBNP was significantly higher in the cardioembolism subtype than in any other subtypes, as assessed according to the TOAST

classification.

CE, cardioembolism; LAA, large-artery atherosclerosis; SVO, small-vessel occlusion; UD, stroke of undetermined etiology
